# Transformable Quadruped Wheelchairs Capable of Autonomous Stair Ascent and Descent

**DOI:** 10.3390/s24113675

**Published:** 2024-06-06

**Authors:** Atsuki Akamisaka, Katashi Nagao

**Affiliations:** Department of Intelligent Systems, Graduate School of Informatics, Nagoya University, Nagoya 464-8603, Japan; akamisaka.atsuki@gmail.com

**Keywords:** wheelchair accessibility, transformable quadruped wheelchair, mobility challenges, stair navigation, robotic legs and wheels integration, reinforcement learning

## Abstract

Despite advancements in creating barrier-free environments, many buildings still have stairs, making accessibility a significant concern for wheelchair users, the majority of whom check for accessibility information before venturing out. This paper focuses on developing a transformable quadruped wheelchair to address the mobility challenges posed by stairs and steps for wheelchair users. The wheelchair, inspired by the Unitree B2 quadruped robot, combines wheels for flat surfaces and robotic legs for navigating stairs and is equipped with advanced sensors and force detectors to interact with its surroundings effectively. This research utilized reinforcement learning, specifically curriculum learning, to teach the wheelchair stair-climbing skills, with progressively increasing complexity in a simulated environment crafted in the Unity game engine. The experiments demonstrated high success rates in both stair ascent and descent, showcasing the wheelchair’s potential in overcoming mobility barriers. However, the current model faces limitations in tackling various stair types, like spiral staircases, and requires further enhancements in safety and stability, particularly in the descending phase. The project illustrates a significant step towards enhancing mobility for wheelchair users, aiming to broaden their access to diverse environments. Continued improvements and testing are essential to ensure the wheelchair’s adaptability and safety across different terrains and situations, underlining the ongoing commitment to technological innovation in aiding individuals with mobility impairments.

## 1. Introduction

In recent years, the number of wheelchair-accessible buildings has been increasing due to the promotion of barrier-free accessibility. However, making some buildings barrier-free remains difficult for a variety of reasons, and it is not easy to completely eliminate stairs and steps. This is a major concern for wheelchair users, and it is equally important to adapt not only the surrounding environment to wheelchairs but also the wheelchairs themselves to the environment.

Quadruped robots can use their four legs to move over a wide variety of terrains, including uneven terrains such as stairs and mountain paths. However, while quadruped robots have high mobility performance, they also have low energy efficiency. This is because the robot is forced to walk even on flat terrain, where quadruped walking is not necessarily required, and because it must constantly supply power to the leg actuators to maintain its posture. Robots with wheels added to the legs [1] have emerged to improve efficiency by using wheeled locomotion. Such innovations have the potential to expand the operational range of quadruped robots and significantly improve their utility.

It has also been found that existing electric wheelchairs that can ascend and descend stairs can only be used to ascend and descend general steps and straight staircases. Therefore, expanding the mobility range of wheelchair users requires the development of a new wheelchair with a mechanism that can be used not only on general stairs but also in a variety of environments.

This research proposes a transformable quadruped wheelchair with a stair-climbing function as a wheelchair that can move along routes with steps and stairs and aims to develop stair-climbing technology through gait control with a passenger. The approach to this research is to first create a simulation environment for a transformable quadruped wheelchair to ascend and descend stairs. This will enable the acquisition of the stair-climbing behavior of a quadruped wheelchair by reinforcement learning. Furthermore, reinforcement learning employing curriculum learning is used to acquire the stair-climbing motion in the presence of a passenger. Based on our findings, we develop a flexible and effective learning method that can handle various scenarios when the quadruped wheelchair climbs over steps and stairs and eventually conduct experiments with a view to using the system in actual equipment.

## 2. Related Work

### 2.1. Stair-Climbing Wheelchairs

There have long been attempts to develop wheelchairs that can ascend and descend stairs. A typical example is the crawler mechanism [2], which consists of multiple wheels connected by a belt. By increasing the contact surface with the ground when traveling, the crawler mechanism can move over uneven terrains, which are difficult for wheels to do. For example, most agricultural machinery uses a crawler mechanism because crawlers with a wide contact surface can travel without sinking even when the soil contains water. The large contact surface also allows the machine to continue traveling even when the crawler locally leaves the ground, which is especially useful in environments with steps or stairs. However, a large contact surface is sometimes a weak point. For example, when rotating in place, the crawler must slide over much of the area in contact with the ground, which reduces stability. Also, in the case of a spiral staircase with different tread lengths on the inside and outside, the left and right crawlers must contact the staircase at different angles. Since most spiral staircases are only wide enough for two people to walk side by side, there is no option to make a large turn so that the angle of the crawlers is more gradual, and the inner crawlers have to contact the spiral staircase at a steep angle, making control difficult.

Quaglia et al. [3] presented Wheelchair.q, a wheelchair that can move in structured and unstructured environments and can climb up and down stairs over obstacles. The wheelchair consists of a frame, a seat, and a four-link mechanism connecting the frame to the seat. When encountering an obstacle, it uses a self-adaptive mobility unit that can passively change its mode of movement from rolling on its wheels to walking on its rotating legs. This mechanism, called a planetary wheel mechanism, is a method in which one large wheel is composed of multiple sub-wheels. The sub-wheels catch on stairs or steps, enabling the wheelchair to ascend and descend stairs. Although this method makes it possible to ascend and descend stairs, it causes a large up-and-down swing when climbing over steps, and it is difficult to ascend and descend spiral stairs.

Thus, there are still many issues to be resolved in the development of wheelchairs that can ascend and descend stairs. By resolving them, it is hoped that wheelchairs that can be used in more environments can be developed.

### 2.2. Quadruped Robots and Their Extensions

Quadruped robots have four legs and walk like dogs, cats, and other quadruped animals. They are rapidly being put into practical use nowadays, especially in places with complex scaffolding such as construction sites. Inverse kinematics is used to derive the control commands for each actuator from the trajectory of the toes to achieve the desired gait. Inverse kinematics is the study of the problem of obtaining the joint angles for the end-effectors in a link mechanism, such as a robot, from the positions and postures of the end-effectors. Inverse kinematics for quadruped robots has been generalized to some extent [4].

Hutter et al. [5] have developed ANYmal [6], a quadruped robot designed for use in harsh environments. Weighing 30 kg and standing 0.5 m tall, it was built in a modular fashion for ease of maintenance and user-friendly handling, with a focus on high mobility and dynamic capabilities. A front- and rear-rotating LiDAR sensor is used for localization and terrain mapping, and compact force sensors on the feet provide accurate measurements of contact conditions. This is the primary reference for the hardware configuration of quadruped robots.

As an extension of quadruped robots, wheeled quadruped robots have been proposed. By combining legs and wheels for improved mobility and versatility, these robots can adapt to real-world environments that require the ability to move quickly and over long distances in complex terrains.

Bjelonic et al. [7] proposed an online trajectory optimization framework for wheeled quadruped robots. The framework partitions the optimization problem into wheel and base trajectory plans that can be solved on-board in real time using a model predictive control scheme, making the robot’s high-dimensional motion planning easier to handle and more robust against unexpected obstacles. They also validated this robot platform in the DARPA Grand Challenge.

Kashiri et al. [8] proposed a wheeled-legged mobile manipulation platform with the necessary strength and mechanical robustness to adapt to real-world applications that require high loads and aggressive physical interaction, such as disaster response scenarios and heavy-lift logistics and coordination tasks. The platform exhibits remarkable physical durability and performance in demanding manipulation tasks while having a body size and weight comparable to that of a human. A high performance is achieved through a combination of design and implementation principles related to the kinematic system, integration of body structure and locomotion, and the wheeled-legged mobility concept. The performance of the robot was tested in a series of experiments that demonstrated its force and strength capabilities in performing tasks involving heavy load manipulation and high-impact physical interaction.

Li et al. [1] presented an extended platform for a high-performance quadruped robot with wheel and leg mode conversion capability. This robotic platform is designed to transport goods in apartments, private homes, and office buildings, and it can move at high speeds using wheels on level ground and legs on other areas such as stairs and wheelchair ramps. In order to achieve a high carrying capacity, a compact torso and leg structure was designed with knee joints and drive wheels. This configuration of wheels installed on a conventional quadruped robot is also adopted in this study.

### 2.3. Gait Control by Reinforcement Learning

Reinforcement learning techniques have made remarkable progress in recent years. Reinforcement learning, one of the key approaches in the field of machine learning, is a method for learning models based on rewards [9,10,11,12]. In reinforcement learning, the control object is called the environment, the controller is called the agent, and the model is based on a Markov decision process [13]. A Markov decision process consists of a state s, an action a, and a reward r. The next state $s_{t+1}$ is determined based only on the current state $s_{t}$ and action $a_{t}$, and the reward $r_{t+1}$ is generated based on this transition. The environment has transition probabilities $p(s_{t+1},r_{t+1}|s_{t},a_{t})$, and the agent learns conditional probabilities from states to actions $\pi \left(a_{t}|s_{t}\right)$ [14].

There are two approaches to acquiring policies by reinforcement learning: the model-based approach, which learns state transition probabilities and determines policies through planning, and the model-free approach, which does not learn state transition probabilities and acquires policies. Model-free methods include the Monte Carlo and temporal difference (TD) methods, which acquire policies indirectly by learning values, and the Actor–Critic method, which acquires policies directly.

In recent years, deep reinforcement learning combined with deep neural networks has achieved significant results, and research is rapidly advancing. DQN [9], which was introduced in 2013, is a TD method that incorporates deep reinforcement learning into Q learning. However, deep learning has made it possible to approximate a huge number of states as a function and thus to treat continuous values such as images and sounds as states. Subsequently, many algorithms based on Actor–Critic appeared [10,11,15] in which policies are learned directly by a neural network. These algorithms made it possible to handle continuous values not only for states but also for actions. With the ability to handle continuous-valued outputs, reinforcement learning has been applied to robots [11]. Simulators have become indispensable for reinforcement learning by robots, and many studies have acquired motion by running robots on simulators and repeating a huge amount of learning [16,17].

There are three general types of gait control methods for quadruped robots: rule-based, learning-based, and hybrid (a combination of rule-based and learning-based methods). The rule-based method manually adjusts the trajectory of the leg tip position, which incurs huge human and time costs. Learning-based methods automatically acquire the trajectory of the leg tip position through reinforcement learning, which is less costly, but there is no guarantee that the desired motion will be acquired. A hybrid method is used for this purpose. In this method, a rough trajectory is determined manually, and detailed fine-tuning is conducted using reinforcement learning [18]. In recent years, many studies have used a hybrid method called PMTG (Policies Modulating Trajectory Generators) [19] or similar ones for quadruped robot gait control [20,21,22,23,24,25]. In the present study, PMTG is employed to fine-tune behavioral parameters through deep reinforcement learning, as described below, to achieve the stair-climbing function.

Hyun et al. [18] implemented a hierarchical controller for advanced dynamic running gait on a quadruped robot called MIT Cheetah. The controller incorporates proprioceptive sensory feedback and compliance with MIT Cheetah’s programmable virtual legs to enable running at speeds of up to 6 m/s (approximately 7.34 Froude number). Three control strategies are employed to achieve a stable and rapid trot gait: programming virtual leg compliance, adjusting stance trajectory, and modulating the gait pattern based on proprioceptive sensory feedback. Based on these strategies, the controller was hierarchically structured and applied to MIT Cheetah in an experimental environment, resulting in a stable trot gait.

Gangapurwala et al. [22] proposed an integrated model-based and data-driven approach in the planning and control of quadrupedal walking for fast travel over uneven terrains. The system uses internal and external feedback to generate a gait plan using a reinforcement learning policy. The system was evaluated using ANYmal, the aforementioned quadruped robot, and auxiliary policies were introduced to deal with different physical parameters and external disturbances. Its applicability to larger and heavier quadruped robots was also demonstrated.

Mimicking the complex behavior of animals has long been a major challenge in robotics. Peng et al. [26] proposed an imitation learning system that allows a quadruped robot to learn agile movement skills by mimicking real-world animals. The system can automatically synthesize controllers with a wide variety of behaviors by utilizing reference motion data and incorporating sample-efficient domain adaptation techniques to rapidly adapt policies learned in simulation to the real world.

### 2.4. Adaptation of Simulation Results to Real-World Environment

Simulations are attractive as training environments as they provide a rich source of data and reduce safety concerns during training. However, robot behaviors developed based on simulator-specific characteristics are often not applicable to their real-world counterparts.

Tobin et al. [27] noted that closing the “reality gap” between simulation and actual hardware experimentation has the potential to accelerate robotics research. Specifically, they focused on a technique called domain randomization, which can train models based on simulated images and transfer them to real images by randomizing the rendering in the simulator. They found that it is possible to train a real-world object detector with an accuracy of 1.5 cm and robustness to disturbing elements and partial shielding by using only data with unrealistic random textures in the simulator.

By randomizing the simulator dynamics during training, Peng et al. [28] developed a policy that is adaptable to a variety of dynamics, including those that differ significantly from the trained dynamics, and showed that it can be generalized to real-world dynamics without any physical training. In an object-pushing task with a robotic arm, their policy, trained only in simulation, can maintain similar performance when deployed on a real robot, reliably moving objects from a random initial placement to the desired location.

Tan et al. [29] proposed a system to automate the design of agile movements for quadruped robots. The system is capable of learning quadrupedal locomotion from scratch from simple reward signals using deep reinforcement learning techniques. Furthermore, the control policy learned by the physics simulator can be deployed to a real robot to improve the accuracy of the physics simulator for real-world applicability and to learn robust policies.

Curriculum learning [30] has been proposed as a method to make reinforcement learning models adaptable to real-world environments, which is achieved by gradually increasing the difficulty of tasks in simulations to a level equal to or greater than the difficulty in the real environment. Curriculum learning is a machine learning approach that elaborates learning procedures to improve learning speed and accuracy. In particular, reinforcement learning requires learning from sparse rewards that contain noise and have time delays, so setting a difficult task from the beginning will cause a phenomenon in which learning does not progress at all. Therefore, a curriculum learning approach is often used because it enables us to guide the search space by designing the learning procedure. In this study, curriculum learning was employed in the learning of the stair-climbing task, oriented toward real-world applications.

Lee et al. [20] presented an innovative, robust foot motor controller for the most challenging environments on earth that are accessible to quadrupeds but inaccessible to autonomous machines. The controller is based on a neural network that is trained within a simulation through reinforcement learning and operates based on its own somatosensory signals. The trained controller retains its robustness for use in natural environments with deformable terrains such as mud and snow, dynamic footholds such as debris, and obstacles such as thick vegetation and violent water currents.

Margolis et al. [31] proposed an end-to-end learning controller with record-breaking agility that can travel and turn at high speeds on natural terrains such as grass, ice, and gravel using the MIT Mini Cheetah. The system sustains speeds of up to 3.9 m/s and responds robustly to disturbances. It is composed of neural networks trained on simulations and uses an adaptive curriculum for velocity commands.

## 3. Transformable Quadruped Wheelchair

### 3.1. Overview

**Figure 1:**
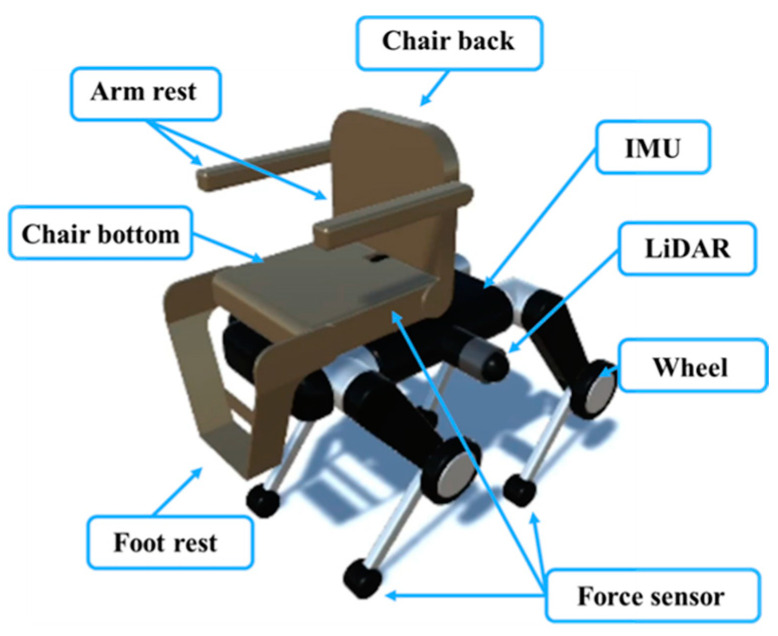
Transformable quadruped wheelchair.

**Figure 2:**
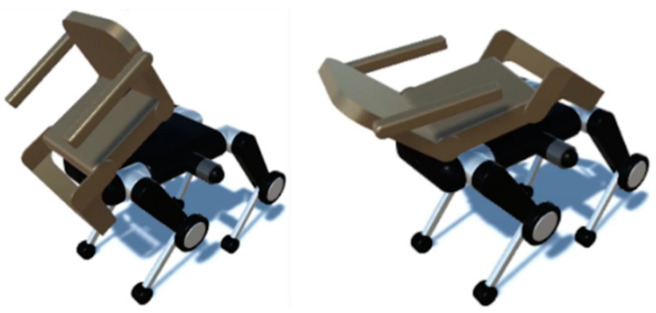
Example of chair transformation.

As shown in Figure 1, the quadruped wheelchair proposed in this study consists of a quadruped-wheeled robot whose lower half is equipped with wheels at the knees and a chair on top of it. The chair is transformable and incorporates actuators in the elbow rest, foot rest, and base. An example of the chair transformation is shown in Figure 2.

The left side of Figure 2 shows the chair tilting forward, while the right side shows the chair reclining backward. This transformation function reduces the shift and sway of the passenger’s center of gravity when ascending and descending stairs, thereby improving gait stability.

**Figure 3:**
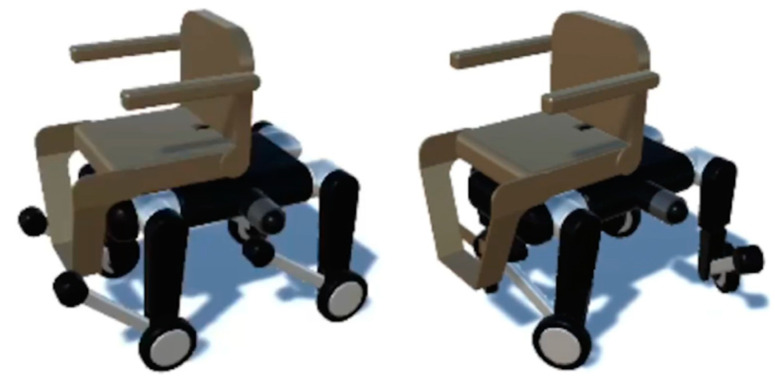
Wheeled transportation.

The lower quadruped-wheeled robot moves by rolling on wheels on flat paths and by quadrupeds on stairs, steps, and other places that cannot be managed by wheels. Figure 3 shows the quadruped wheelchair moving on wheels. Not only can the wheelchair move forward and backward, but by rotating the legs and adjusting the direction of the wheels, it can also make a counter-rotation turn and move parallel to the left and right. The quadruped wheelchair proposed in this paper was designed with reference to the Unitree B2 [32] developed by Unitree Robotics (Hangzhou, China) described below. Since the wheeled model (Unitree B2-W [33]) has a maximum range of 50 km (with a load of about 40 kg), the proposed quadruped wheelchair is assumed to have a similar range with a human on board.

Since control during gait is based on information from the outside world, the robot is equipped with multiple sensors. Specifically, hemispherical 3D LiDAR sensors are installed on either side of the quadruped-wheeled robot’s body, an IMU is attached to the body, and force sensors are attached to the toes. These sensors allow the wheelchair to accurately assess its surroundings for safe and efficient mobility.

**Figure 4:**
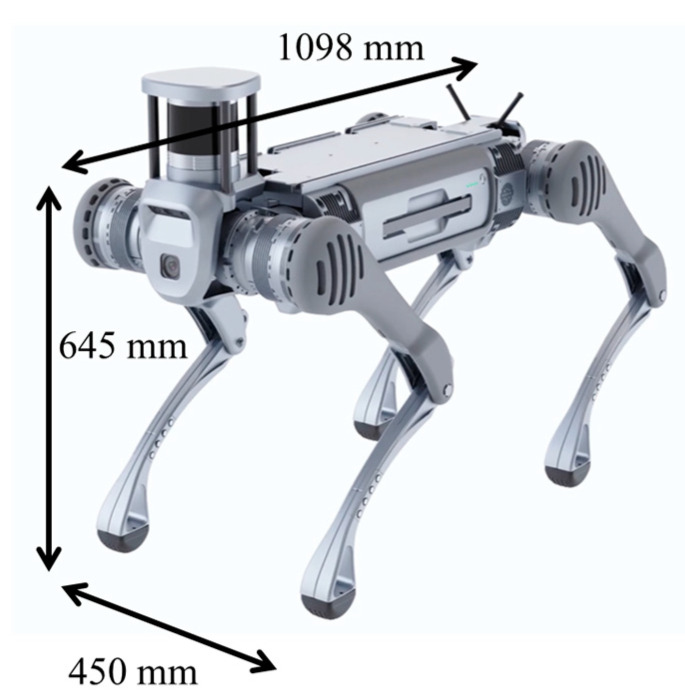
Dimensions of Unitree B2.

**Figure 5:**
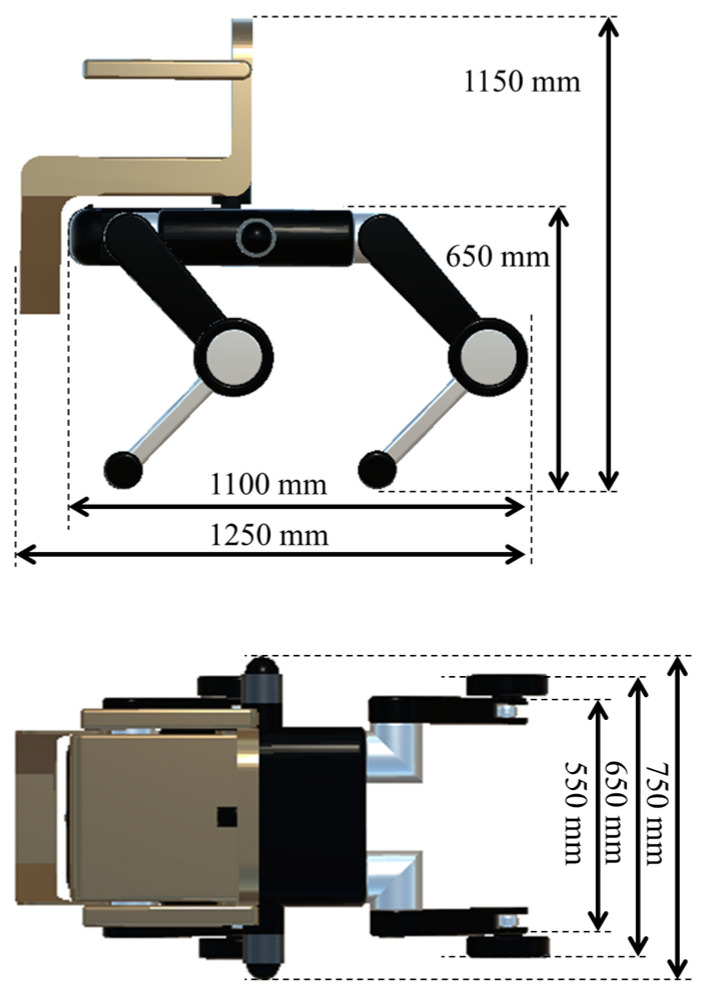
Dimensions of transformable quadruped wheelchair (top, vertical; bottom, horizontal).

Figure 4 and Figure 5 show the dimensions of the commercially available quadruped robot Unitree B2 and the proposed transformable quadruped wheelchair, respectively. Because of the chair and wheels, the overall dimensions of the quadruped wheelchair are slightly larger than those of Unitree B2; however, if the chair is removed, the basic body dimensions are almost the same. The type of sensors required was determined with reference to existing research on self-built quadruped robots [34,35,36,37].

**Figure 6:**
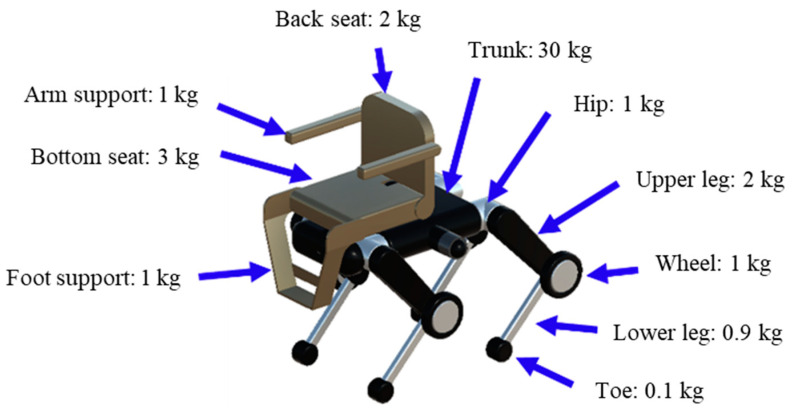
Weight setting of various parts.

Since the masses of the various parts play a very important role in simulation experiments, the values were determined by referring to the Unitree B2 URDF file officially published by Unitree [38]. The URDF (Unified Robot Description Format) file is an XML format for describing the structure of a robot. The contents of the URDF file include the loading positions of the 3D models of the parts that make up the robot, rotation, weight, friction force, actuator parameters, and sensor information, which are important for knowing the details of the robot’s internal structure. The weight of each part, which was determined using this information, is shown in Figure 6. The trunk weighs 30 kg, and the weights of the leg parts are, from top to bottom, 1 kg for the hip, 2 kg for the upper leg, 1 kg for the wheel, 0.9 kg for the lower leg, and 0.1 kg for the toe. As for the chair portion, the back seat weighs 2 kg, arm support 1 kg, bottom seat 3 kg, and foot support 1 kg.

### 3.2. Hardware Configuration

This subsection describes the mechanism and role of each joint of the transformable quadruped wheelchair. This wheelchair has twenty joints in the quadruped-wheeled robot part and seven joints in the chair part. Since the leg structure of the quadruped-wheeled robot is basically the same in the front and back and on the left and right, we will focus on the structure of the left front leg as a concrete example.

**Figure 7:**
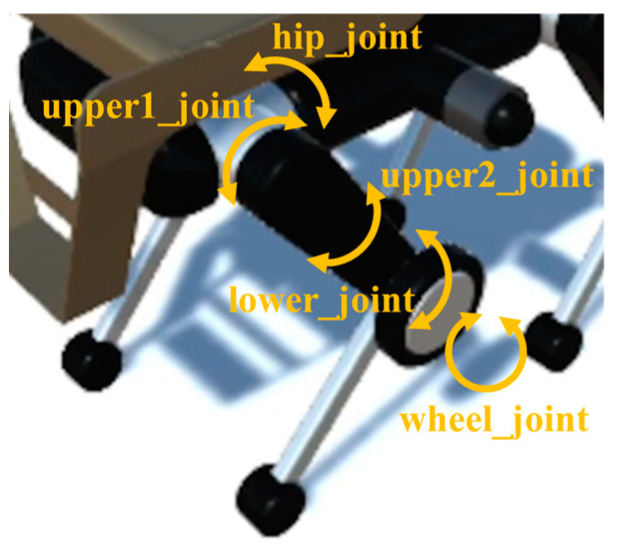
Left front leg joint.

As shown in Figure 7, the left front leg has five joints, which from the top are the hip_joint, upper1_joint, upper2_joint, lower_joint, and wheel_joint. These names are from the URDF in the ROS package for quadruped robots. The hip_joint is located at the top of the leg and is responsible for the left/right rotation function, which is important when walking and during balance adjustment. The upper1_joint follows the hip_joint and has the function of swinging the leg back and forth, which is essential for walking. The upper2_joint is located below it and is mainly responsible for changing the direction of the wheels during wheel movement. The lower_joint has the function of swinging the lower half of the leg back and forth, which is important when walking. The wheel_joint is coaxial with the lower_joint and is used during wheel movement.

**Figure 8:**
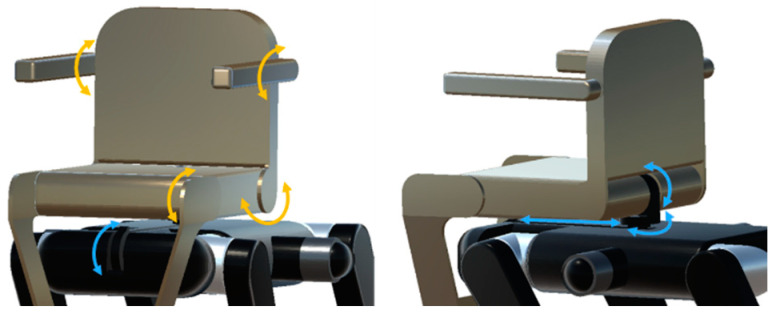
Chair armrest and body joints.

Next, the joints of the chair are described. The chair is coupled to the body of the quadruped-wheeled robot and has multiple degrees of freedom (DOFs). In Figure 8, the chair arm joints are shown in blue and the DOFs of the chair body are shown in orange, each numbered. Looking at the chair arm joints, joint (1) is for tilting the chair forward and contributes to the function of keeping the passenger level when ascending or descending stairs. Joint (2) is a slider mechanism that slides the chair forward and backward, allowing the passenger’s center of gravity to move back and forth. Joint (3) rotates the chair to the left or right, which helps to adjust the orientation of the wheelchair and the passenger when ascending or descending stairs. Joint (4) tilts the main body of the chair backward. As for the joints of the chair body, (5) adjusts the tilt of the backrest, (6) adjusts the tilt of the footrest, and (7) and (8) adjust the angle of the armrests. Appropriate control of these chair joints can minimize changes in the position and moments of the passenger’s center of gravity when ascending and descending stairs and can contribute to improving the stair-climbing performance of the quadruped wheelchair.

Several sensors are essential for a quadruped wheelchair to acquire proper gait motion. In particular, 3D LiDAR, inertial sensors, and force sensors play an important role in the environmental awareness of the quadruped wheelchair. Since an actual machine does not exist at this time, the functions of these sensors are assumed and their parameters are set in modeling and simulation.

LiDAR (light detection and ranging) measures distance by illuminating an object with light and detecting the reflected light. This technology is often used by autonomous mobile robots for self-position estimation. However, in this research, LiDAR is not used for that purpose, but for recognizing foothold conditions; it is installed on both sides of the body of a quadruped wheelchair and is modeled after the commercially available RS-Bpearl [39] from RoboSense Technology Co., Ltd. (Shenzhen, China). The simulations reproduce the sensor behavior based on the measurable range, resolution, and response frequency of RS-Bpearl.

An inertial sensor, commonly known as an inertial measurement unit (IMU), includes an accelerometer and a gyroscope and is used by a robot to determine its own motion state. Accelerometers are used to measure the acceleration of an object, especially with respect to linear motion. Gyroscopes, on the other hand, detect the rotational motion of an object and measure its angular velocity. These sensors are widely used in smartphones, tablets, automobiles, aircraft, and robots. As for inertial sensors, there is no clear reference product like LiDAR, but we programmatically reproduced a six-axis sensor with an accelerometer and a gyroscope. This allows the quadruped wheelchair to use its own tilt and acceleration information to process the risk of tipping over and the magnitude of the sway.

A force sensor is a device that detects forces applied to an object and measures their magnitude and direction. These sensors are widely used to accurately measure forces acting on machines and structures and play an important role in fields as diverse as industry, robotics, the automotive industry, and medical technology. Force sensors sense forces using various principles, such as the piezoelectric effect and changes in resistance and capacitance. Force sensors of particular interest in the field of robotics are those that can measure force as a three-dimensional vector, and these sensors can be used to detect force components along the X, Y, and Z axes separately. This capability allows both the direction and magnitude of the force to be measured simultaneously, making it possible to handle more complex force changes. This type of force sensor is particularly useful in applications requiring precise force and motion control, such as sophisticated robotic arms, precision industrial equipment, human motion analysis, and devices requiring haptic feedback. The measurement of three-dimensional forces also contributes to more precise control and manipulation of objects, leading to increased safety and efficiency. In this study, a force sensor was used to measure the contact force of the toes as a three-dimensional vector.

## 4. Control of Quadruped Robot Gait

### 4.1. Overview

The quadruped gait is characterized by its dynamic stability and mobility, especially on irregular terrains, and is designed to mimic the quadrupedal gait of living animals, with each leg moving alternately or diagonally to maintain equilibrium. This gait pattern is closely coordinated with the movements of the other legs, rather than each leg operating independently, and their interaction optimizes overall balance and efficiency.

The movement of each leg in the quadruped mechanism has a direct impact on the stability and mobility of the robot as a whole. For example, while one leg is in contact with the ground, the other adjusts its position for the next step. In this way, the movements of each leg work together seamlessly to achieve a smooth and natural gait. In addition, the gait control of the quadruped mechanism requires high mobility in each joint of the legs because of the need for adaptability to uneven terrains. This enables the robot to move efficiently over complex terrains, such as rocky and bumpy terrain.

There are many different gait patterns for the quadruped robot, including the walk, trot, and gallop. Each of these gait types has different properties and is selected appropriately for the situation. The walk is the most basic form of gait, in which only one leg is moved at a time. It is a very simple method and does not require the implementation of complex controls, making it easy to implement. The trot is characterized by the simultaneous movement of the diagonal legs and is faster than the walk. The gait method of dogs, cats, and other animals is basically a trot. This method of gait is suitable for moving at moderate speeds due to its repeated movements in a steady rhythm, and it is commonly employed in many quadruped mechanisms. The gallop is a gait pattern used when faster movement is required, in which all of the robot’s legs temporarily leave the ground. This dynamic and powerful gait is useful in situations where high-speed movement is required or for quickly traversing more difficult terrains.

**Figure 9:**
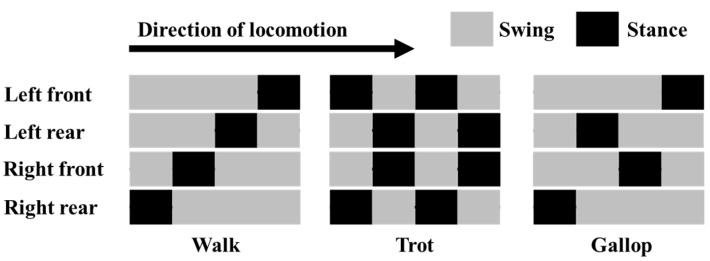
Typical gaits of a quadruped mechanism.

The timing of leg swing and stance for these three gait types is represented in Figure 9, where LF stands for left forelimb, LH for left hindlimb, RF for right forelimb, and RN for right hindlimb. Here, swing means that the toes are not in contact with the ground, and stance means that the toes are in contact with the ground.

### 4.2. Trajectory Generator

A trajectory generator (TG) is essential for defining the gait pattern of a quadruped mechanism. This device or algorithm plans when and how the toes of the quadruped mechanism will make contact with the ground and how it will move. Accurate trajectory generation plays a central role in optimizing energy efficiency, stability, and flexibility. The ability to dynamically adjust trajectories in response to changes in the environment also enhances the adaptability of the robot. This subsection describes the basic purpose of the TG, how it works, and how it affects the overall performance of the quadruped robot.

The TG is a mechanism for defining the motion paths for the endpoints of a robot, for example, the hand of a robotic arm or the toe of a walking robot. In particular, in a quadruped mechanism, it is responsible for generating motion paths for each of the four legs.

**Figure 10:**
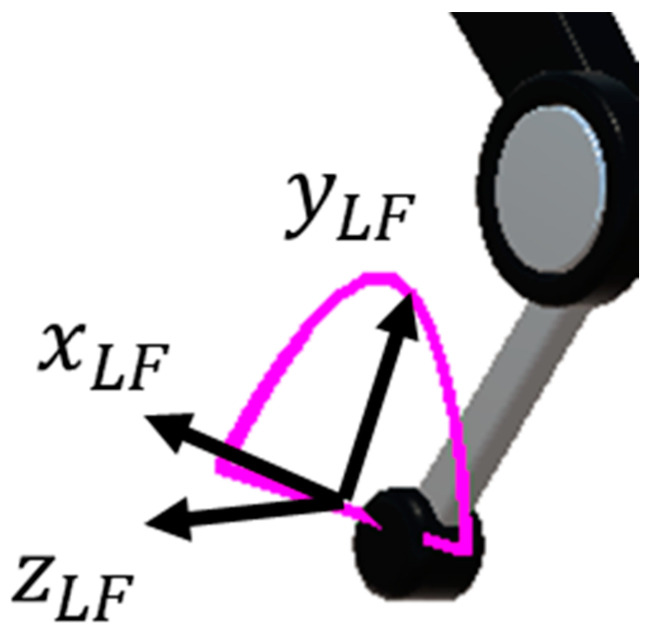
An example of trajectory generated by the TG.

Figure 10 shows an example of a trajectory generated by the TG for a quadruped mechanism. In this figure, a trajectory with a flat bottom and rounded top is drawn for the toe area. This is called the “trajectory,” and the origin of its coordinate system is placed at the reference position of the toe. Importantly, the appropriate trajectory varies with the terrain and environment, necessitating a rotation of the trajectory’s local coordinate system and fine-tuning of the trajectory’s output. This flexibility allows the quadruped mechanism to move efficiently over a wide variety of terrains and conditions. The purple line in the figure represents the trajectory generated by the TG as it is, and the PMTG described below uses this trajectory as a base and adds real-time fine-tuning to it to create the actual trajectory to be executed.

In a quadruped mechanism, each of the four legs has an independent TG. These generators are designed to allow the robot to change its gait in various ways, and the synchronization of motion frequency is an important factor.

There are many toe trajectory generation methods, each with their advantages and disadvantages. In this subsection, the three main different trajectory generation methods will be described.

Periodic TGs are specialized for gait movements that repeatedly execute a specific rhythm or pattern. This type of TG generates movements with a certain periodicity, thus enabling a stable gait for the quadruped mechanism. The periodic trajectory generation approach uses basic mathematical formulas and algorithms that generate repetitive gait cycles. This approach is particularly well suited for walking on flat ground with little variation and allows for a relatively stable gait even without sensors or sophisticated control techniques. The following is a brief description of the sine wave-based TG. The TG using a sine wave is represented by the following equations:

(1) $$x\left(t\right)=\frac{w}{2}\cos\left(\phi \left(t\right)\right)$$

(2) $$y\left(t\right)=\left\{\begin{array}{cc} 0, & 0\leq \phi \left(t\right)<\pi \\ h\;sin(\phi \left(t\right)-\pi ), & \pi \leq \phi \left(t\right)<2\pi \end{array}\right.$$

(3) $$\phi \left(t\right)=mod\left(\phi_{0}+2\pi \left(f+f_{offset}\right)t,\;2\pi \right),$$

where x(t) and y(t) are the x-coordinate and y-coordinate at time t, and the coordinates of the toe on the two-dimensional plane are expressed as $\left(x\left(t\right),\;y(t)\right)$. Equation (1) represents the expression for the value of the x-coordinate, which is the value obtained by applying a cosine to the function ϕ that converts time information into angle information defined as in Equation (3), multiplied by w/2, where w is the width of the TG. Equation (2) represents the expression for the value of the y-coordinate, conditioned by the value of ϕ. The value of the y-coordinate is 0 when $0\leq \phi \left(t\right)\leq \pi$ because it is stance, and the value of $hsin(\phi \left(t\right)-\pi )$ is adopted when $\pi \leq \phi \left(t\right)\leq 2\pi$ because it is swing so that a sine curve is drawn, where h is the height of the TG.

The TG using a sine wave is the simplest one, and there are other TGs such as spline and Bezier trajectories.

Learning-based TGs aim to generate optimal gait trajectories by learning and extracting optimal gait patterns from large amounts of data using machine learning technology. This learning-based approach is challenging because the design of the learning environment has a significant impact on the performance of the acquired TG. A representative approach is the Policies Modulating Trajectory Generator (PMTG) [19], which is described in detail in the next subsection.

### 4.3. Policies Modulating Trajectory Generators

PMTG is a method for optimizing gait behavior by fine-tuning the parameters and output of TGs with measures. In this approach, the policies and TGs complement each other to mitigate the difficulty in acquiring gait behaviors by relying on policies alone and the difficulty in generating optimized trajectories for the environment by relying on TGs alone.

The advantage of this approach is that it combines rule-based and machine-learning-based approaches to simultaneously increase the accuracy and flexibility of gait control. While TGs provide efficient gait patterns in predictable and general environments, such as walking on flat surfaces, they have limitations in dealing with complex environments, such as those with a wide variety of steps. On the other hand, measures based on machine learning have the ability to flexibly adapt to these complex situations.

**Figure 11:**
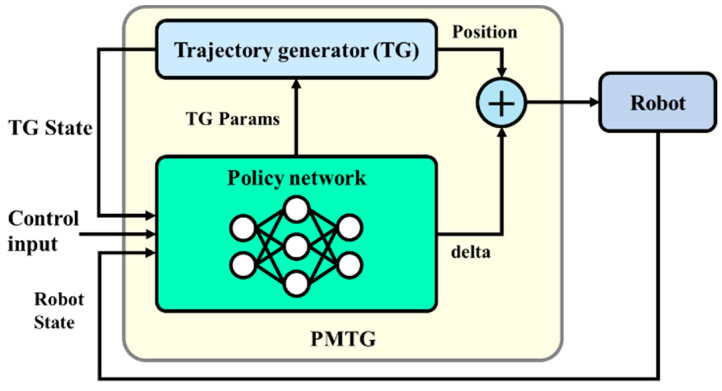
PMTG structure.

The specific mechanism of PMTG is shown in Figure 11. The left-hand area of Figure 11 describes the TG and the policy. The arrows from the policy to the TG indicate the adjustment of parameters such as its width and height. In the ⊕, the policy makes minor adjustments to the output of the TG. The inputs to the strategy include information from the controller, such as the direction of travel, state information, such as sensor data from the robot, and the state of the TG. Although the structure is relatively simple, this mechanism can be used for reinforcement learning to obtain a walkable trajectory even in complex environments.

In designing the PMTG, the first step is to select a base trajectory generation algorithm. Possible choices include sine, spline, and Bezier trajectories. Although the shape of these trajectories may differ somewhat, they are not expected to have a significant impact on the trajectories after learning, as they will eventually be fine-tuned by the policy. If there is no particular preference, it is common to select a sinusoidal trajectory.

The next step is to determine the inputs to the measures in the PMTG. This process determines what numerical values, such as sensor data or controller values obtained from the robot being simulated, will be used as inputs. It also determines whether to treat them as sequential data or not. Finally, the structure of the neural network that will learn the measures is determined. Typically, a multilayer perceptron (MLP) is used, but a temporal convolutional network (TCN) [40] can also be used if the input is sequential data. Since the multilayer perceptron has a low functional bias, it is likely to achieve reasonable accuracy after a certain amount of time. It is best to try the multilayer perceptron first, and then try different architectures if we need to achieve even higher performance.

**Figure 12:**
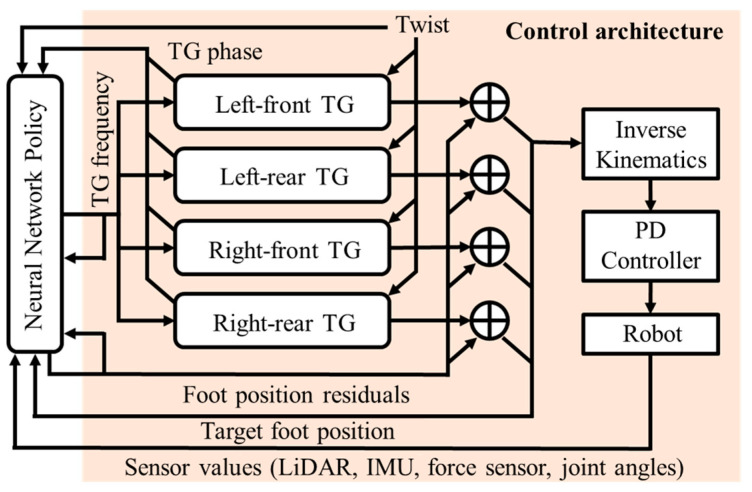
Structure of PMTG used in this research.

Figure 12 depicts the general concept of the PMTG shown in Figure 11 and illustrates the structure used in this study. In Figure 12, independent TGs are provided for each leg (left foreleg, left hind leg, right foreleg, and right hind leg) for a total of four TGs. These TGs are realized as sinusoidal trajectories, and fine-tuning is also performed by a single neural network. The TG Params in Figure 11 become TG frequency in Figure 12, and control input is embodied in Twist. In addition, the robot state is embodied in LiDAR, IMU, force sensor, and joint angles; foot position residuals and target foot position are described below.

In reinforcement learning of the PMTG, learning is performed in the area described as the “neural network policy” in Figure 12. Since it is necessary to know the internal state of the TG when learning, the information received by the TG and its output information are received as input. Specifically, the input data to the policy network includes a wide range of information such as foot position residuals, target foot position, LiDAR, IMU, force sensor, joint angles, TG frequency, TG phase, and Twist. Foot position residuals represent the amount of fine-tuning made to the previous TG output and are so named because they are similar to ResNet’s Residual Connection [41]. The target foot position is the final output value of the PMTG, which is the output of the TG plus the foot position residuals. The LiDAR input is the 3D information of the acquired point cloud, the IMU input is the acceleration and angular velocity data, and the force sensor input is the force sensor data from the four toes. The force sensor values obtained from the four toes are received from the force sensor as a 3D vector. TG frequency indicates the operating frequency of the rule-based TG and is used to adjust the speed of leg movements according to the situation. It functions as a type of state information for the TG. It is also designed to receive Twist information, which indirectly allows the TG to obtain the yaw angle at which the TG is set to move in any direction.

The policy network uses the above information to perform reinforcement learning and calculate the output of the PMTG, which is passed to the program for solving the inverse kinematics and converted into target joint angle data. The actuators are then operated via the PD controller to achieve the robot’s gait.

**Figure 13:**
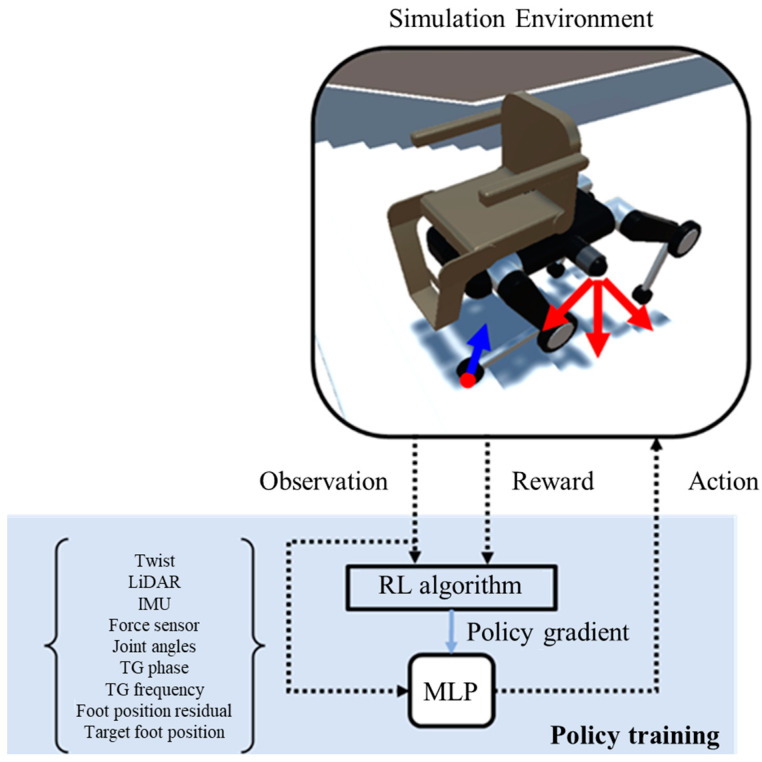
Reinforcement learning for optimization of policy network.

Figure 13 shows a schematic diagram of the optimization of the neural network policy (specifically, the MLP in this research) in Figure 12 by a reinforcement learning algorithm. The reinforcement learning algorithm optimizes the MLP by using the policy gradient-based or Actor–Critic method to optimize the continuous-valued behavior. Although the PMTG has a very complex structure, the integrated reinforcement learning problem boils down to the general reinforcement learning problem of optimizing a policy network that outputs fine-tuned values for a TG.

## 5. Simulation Environment for Quadruped Wheelchair

### 5.1. Implementation

Unity [42] is a game engine developed by Unity Technologies (San Francisco, CA, USA) widely used to create 3D content, and it was used as the primary tool to build the simulation environment for the quadruped wheelchair in this study. Unity’s real-time 3D rendering capabilities allow for the real-time visualization of the robot’s behavior and the physical interaction of the environment, creating a realistic simulation environment for testing. Unity’s advanced physics engine can also simulate physical phenomena such as gravity, friction, and collisions with a high degree of accuracy, making it possible to reproduce on a computer the various situations that a quadruped would encounter in a real environment and analyze its behavior. The version of Unity we used is 2020.3.17f1.

Other robot simulation environments exist, such as Gazebo [43], which is a ROS standard, and PyBullet [44], which runs on Python; Gazebo works well with ROS, but it is not as good as Unity as a simulation environment. PyBullet is also designed to run in Python and is easy to integrate with learning programs such as PyTorch, but it does not have the advanced editor features of Unity. Unity allows for the detailed adjustment of visual elements such as robot models, textures, and light settings for the environment, and can simulate the appearance and behavior of the robot in a form similar to that of the actual robot, as well as perform machine learning at a high speed.

Another important feature of Unity is its extensibility and versatility: it supports a wide variety of plug-ins and APIs, which facilitate integration with machine learning algorithms and other external tools. Specifically, ML agents [45], described below, can be used to perform advanced machine learning at a high speed, and the Unity Robotics Hub [46], described below, can be used for integration with ROS. This makes it possible to conduct simulation experiments under a variety of scenarios and conditions prior to integration testing with actual robot hardware in the development of motion control algorithms for quadruped wheelchairs.

### 5.2. Creating a Quadruped Wheelchair with Unity

In order to perform a stair-climbing experiment on a quadruped wheelchair, its actuators and sensors need to be simulated. To simulate the actuators, the ArticulationBody component of Unity was used. This component eliminates the inaccuracies of previous joint simulations and allows for more accurate simulations. See Appendix A for details on how to set the key parameters.

For the sensors, a hemispherical 3D LiDAR, an IMU, and a force sensor were used. These sensors play an important role in the motion analysis of the quadruped wheelchair. See Appendix B for details on how LiDAR was set up and how the other sensors were implemented.

### 5.3. Direction Control by Navigation System

In this research, we envision a system that automatically ascends and descends stairs. This system is realized by two mechanisms: automatic path generation and determination of the direction of travel and gait control according to the path. For path generation and direction determination, the NavMesh component [47] provided officially by Unity is used. The gait control part is achieved by modifying the TG based on the PMTG described in Section 4.3.

**Figure 14:**
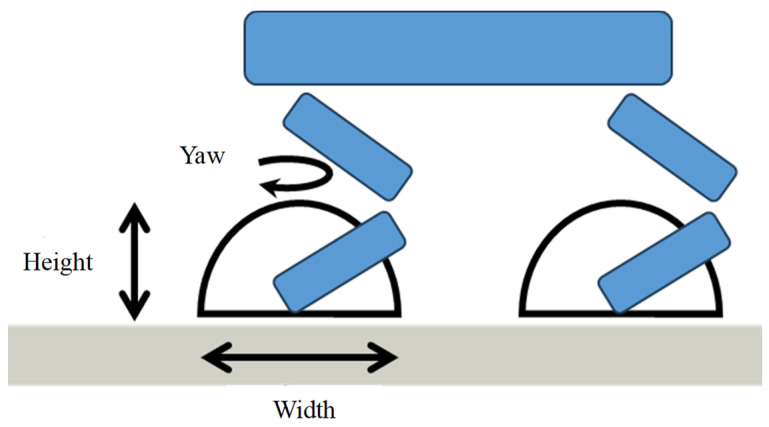
Width, height, and yaw angle of TG.

In this research, a mechanism was used to dynamically change the direction of the TGs by utilizing the direction-of-travel data computed using NavMesh. The TGs set at each toe can change their width, height, and yaw angle parameters. As shown in Figure 14, direction-adaptive gait is achieved by adjusting these parameters individually using the direction-of-travel data obtained from NavMesh.

**Figure 15:**
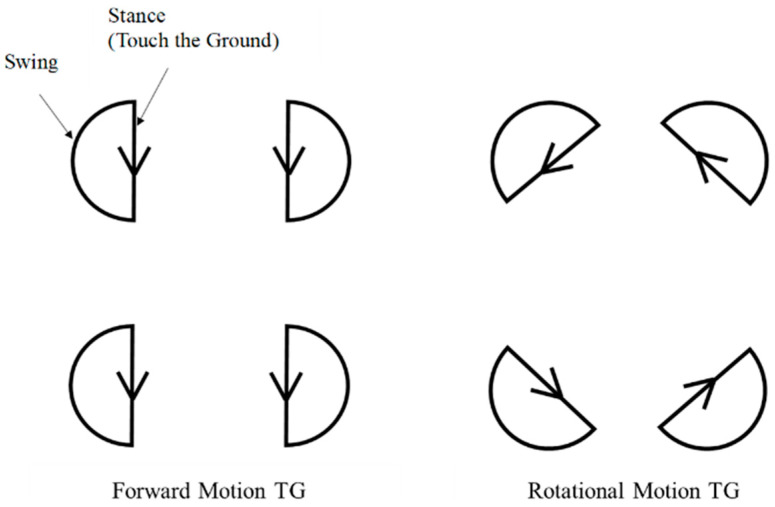
TG for forward/backward and rotational movements.

Specifically, there should be two sets containing four TGs. As shown in Figure 15, one set generates trajectories associated with the forward/backward movement (the yaw angle of this set is 0), and the other set generates trajectories associated with the rotational movement (the yaw angle of this set is 45).

**Figure 16:**
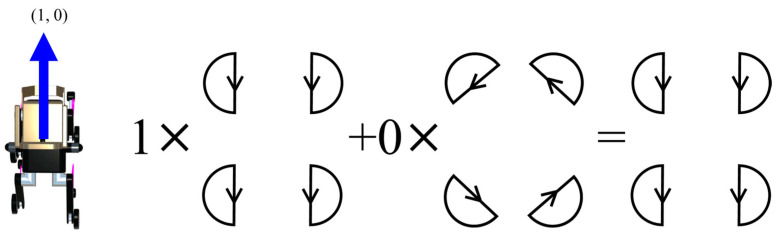
The output trajectory of the final TG when the direction of travel is straight ahead (1, 0).

**Figure 17:**
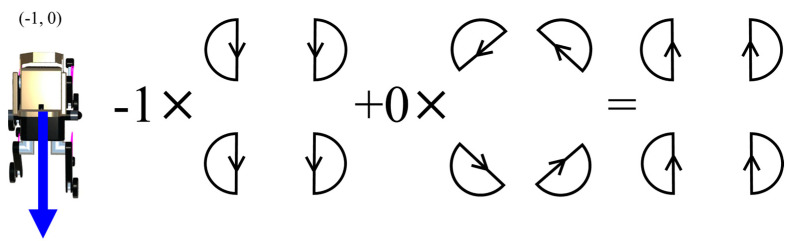
The output trajectory of the final TG when the direction of travel is backward (−1, 0).

**Figure 18:**
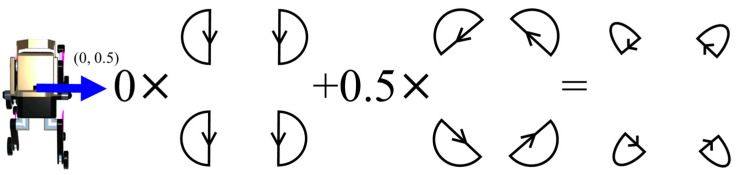
Output trajectory of the final TG when the direction of travel is right (0, 0.5).

**Figure 19:**
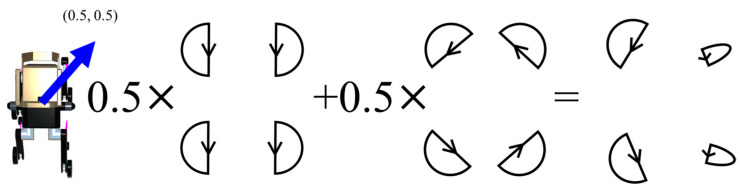
The output trajectory of the final TG when the direction of travel is diagonally right (0.5, 0.5).

The direction-of-travel data obtained from NavMesh is expressed in a two-dimensional coordinate system with respect to the robot’s torso, which includes a forward/backward and a left/right component. Therefore, the final output of the TG is the sum of the output of the TG that controls the forward/backward movement multiplied by the forward/backward component of NavMesh and the output of the TG that controls the rotation direction multiplied by the left/right component of NavMesh. Figure 16, Figure 17, Figure 18 and Figure 19 show specific examples.

Figure 16 shows the calculation for the case where an input is received in the straight direction [in coordinates, (1,0)]. In this case, 1 is multiplied by the TG that controls the forward/backward movement and 0 is multiplied by the one that controls the rotational movement, so the final trajectory is the output of the TG that controls the forward/backward movement.

Figure 17 shows the calculation for the case of a backward trajectory directly behind (−1, 0). Here, the direction of rotation of the trajectory is reversed because the sign is negative. As a result, the output of the TG, which governs the backward and forward movement, is in the opposite direction, which is the final output.

Figure 18 shows the case where the direction of travel is directly right (0.5). In this case, the TG governing the forward/backward movement is multiplied by a factor of 0, and the TG for the rotational direction is multiplied by 0.5. This results in an output with a width of 0.5 times the width of the TG governing the movement in the rotational direction, resulting in half the rotational speed.

Figure 19 shows a diagonal rightward (0.5, 0.5) progression. In this case, 0.5 is multiplied by the TGs that control the forward/backward movement and the rotational movement. As a result, the left leg makes a large left rotation and the right leg makes a small right rotation, and the robot progresses in the diagonal right direction.

These calculations allow the TG to reach its destination alone, at least in flat environments, by applying NavMesh’s path calculation and direction-of-travel derivation to the TG calculations.

### 5.4. Overview of Simulation Environment

**Figure 20:**
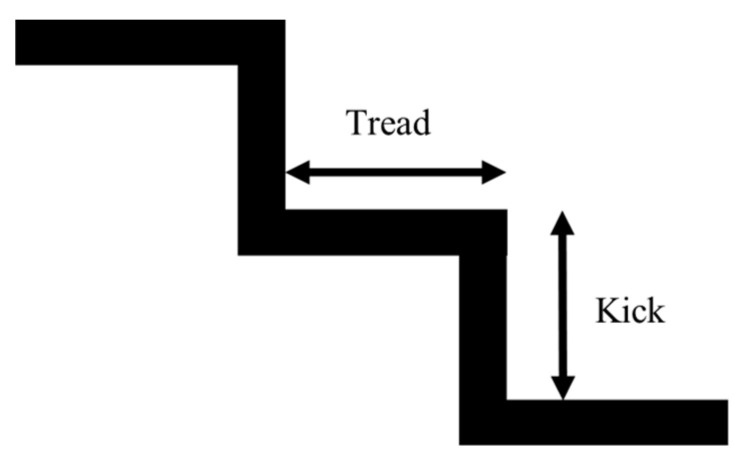
Staircase kick and tread.

The setting of the staircase in the simulation is an important element in this research. This subsection describes the standards of the staircase. The most important aspects of staircase design are the kick and the tread. The kick height refers to the height of each step of the staircase, and the tread refers to the depth of the flat portion of the staircase as shown in Figure 20. These sizes are defined by the Building Standard Law.

In a typical house, the kick height is considered to be 23 cm or less and the depth of the treads to be 15 cm or more. However, public facilities require even more accessible designs, and it is common for the kick height to be 15 cm and the tread depth to be 30 cm. Thus, staircases in public facilities are designed with a lower kick height and wider tread depth. However, there is no legally defined lower limit for the height of the kick. For example, when small children or the elderly are considered, it may be appropriate to set the kick height at about 12 cm.

**Figure 21:**
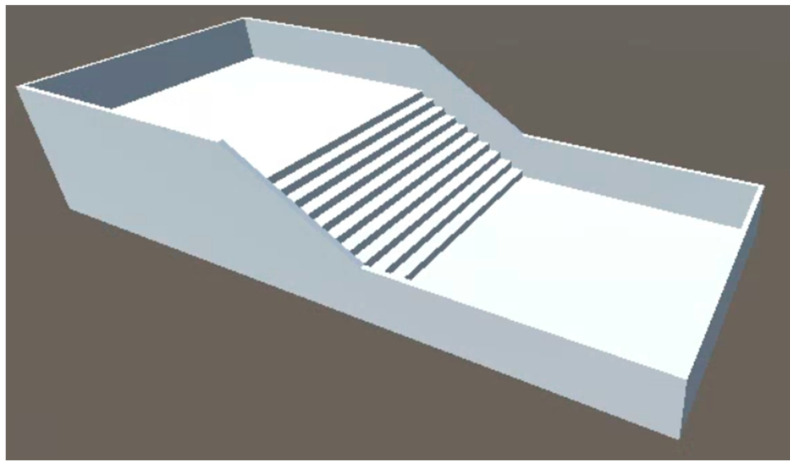
A 3D model of the staircase.

Thus, the kick height and tread depth vary depending on the intended use of the building and the target audience. In this research, the kick height of the staircase was set to 12 cm for a staircase that can be easily used by all people, assuming that the staircase is a public facility. The 3D model used in the experiment was created in CAD and loaded into Unity as shown in Figure 21. To prevent the user from moving out of the environment and falling during the experiment, a wall was placed around the staircase. The number of steps is ten.

In this research, the Tesla Bot is used as the passenger model. The Tesla Bot is a humanoid robot developed by Tesla, Inc. that is 173 cm tall, weighs 57 kg, and can walk at a maximum speed of about 8 km/h. It is also capable of carrying a 20 kg load. The Tesla Bot is characterized by the fact that it was developed to look more human-like than previous humanoid robots. A 3D model of the Tesla Bot is used in the simulation in this study, but the actual device is not yet available.

Since the purpose of this research was to perform simulations assuming a person sitting in a wheelchair, a model that is extremely close to human geometry was needed. The Tesla Bot can meet this requirement; the 3D model of the Tesla Bot [48] is publicly available, and this 3D model was loaded into the Unity simulation environment for this study and used in the experiments.

**Figure 22:**
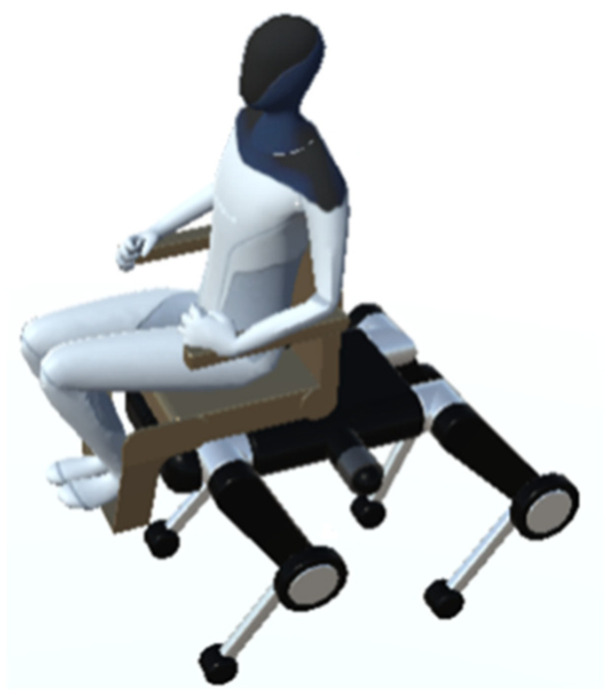
Tesla Bot in quadruped wheelchair.

The Tesla Bot is shown in Figure 22 boarding a quadruped wheelchair. Since the model at the time of release was in an upright position, each body part was appropriately rotated and adjusted to a sitting pose. 

## 6. Stair-Climbing Experiment

### 6.1. Experiment for Acquiring Behavior in Staircase Ascent

**Figure 23:**
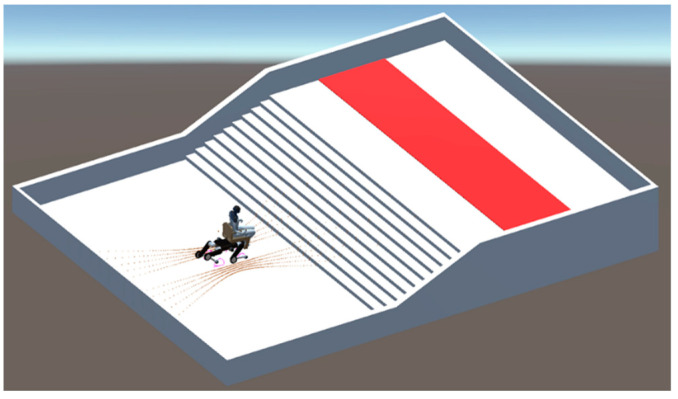
Field used in stair-ascending motion acquisition experiment.

A CAD-designed staircase environment was used in the stair-ascending motion acquisition experiment as shown in Figure 23. The path calculated using NavMesh was designed to head toward the center of gravity of the goal area. In the experiment, PMTG was used for gait control and Proximal Policy Optimization (PPO) [10] was employed as the reinforcement learning algorithm. Instead of tackling this complex task directly, an appropriate curriculum will be set up to make the goal reachable.

In this research, curriculum learning is employed to obtain measures that can eventually be applied to tasks in the learning environment by adjusting the difficulty level of the task in stages. In this approach, it is essential to select parameters to be varied according to learning progress and to formulate a procedure for changing them.

The main factors selected to adjust the difficulty level in learning stair climbing were the height of the stair kicks, the distance to the goal, and the passenger’s body weight. The height of the stair kicks and the distance to the goal were selected for the following reasons: In reinforcement learning, random actions are often taken in the initial stages. This is because the weights of the measures are determined randomly at the beginning and are in a high entropy state, which increases the randomness. Thus, the initial quadruped wheelchair will have difficulty even moving forward and even more difficulty reaching the goal, especially if the kicks are high or the goal is far away. The reasons for selecting the passenger’s weight are as follows: Although learning proceeds along the motion defined by the TG, it is difficult to obtain a stable gait that reaches the goal only with the TG in a tuned state without a passenger, because the pattern of motion changes as the passenger rides on the TG. With a passenger on board, the risk of falling increases with only the TG, which may hinder the progress of the curriculum. Therefore, we decided to start with a low passenger weight and increase it gradually.

For these reasons, the height of the kicks, the distance to the goal, and the weight of the passenger were selected as the main modification parameters for the curriculum and adjusted within the ranges shown in Table 1. The curriculum starts with these elements at their minimum values and gradually increases them to their maximum values.

**Figure 24:**
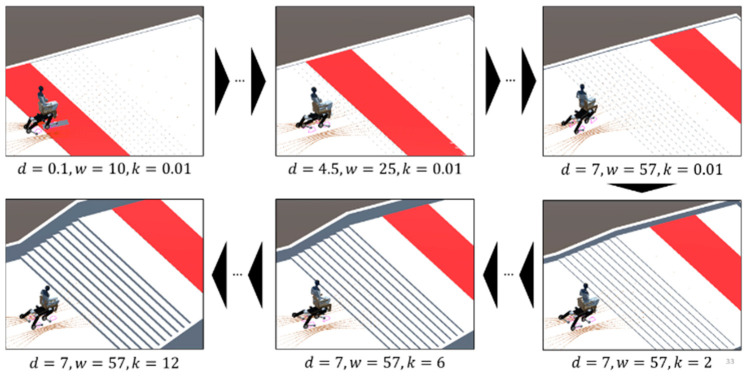
Curriculum changes.

The process of change in this curriculum is shown in Figure 24, where the variable d represents the distance to the goal, w represents the passenger’s weight, and k represents the kick height. In the initial stage of learning, the goal position is very close to the quadruped wheelchair, with a passenger weight of 10 kg and almost no kick height. From this stage, the approach is to increase the passenger’s weight while gradually moving the goal position away from it.

Although there were three parameters to be varied by the curriculum in this experiment, whether it is possible to change multiple parameters simultaneously is an important aspect of curriculum design and is therefore explained here. The criterion for determining whether simultaneous changes are possible is whether each element to be changed can be considered independent of each other. For example, if the goal position and kick height are increased simultaneously, the kick rise will not make sense if the goal position is not over the staircase when the kick rises. In other words, the goal may be reached before the staircase is reached and the episode may be terminated. On the other hand, even if the learning progresses and the goal position reaches the staircase, the intermediate curriculum on the kick rise will be skipped.

In contrast, with respect to weight, learning to move the goal position farther away and learning to increase weight can be conducted simultaneously, and one curriculum does not limit the other. Thus, the curriculum of increasing weight and moving the goal position away from the goal can be implemented simultaneously. In this way, the distance to the goal and body weight are designed to reach their maximum values at the same time, while the kick height remains at its minimum value. After the distance to the goal and body weight have reached their maximum values, only kick height is gradually increased.

### 6.2. Reward Function

The reward function plays an important role in the acquisition of stair-climbing behavior by the quadruped wheelchair. The formulas and rationale for each reward item will be explained in detail.

#### 6.2.1. Immediate Reward

The immediate reward r is calculated by summing several reward factors. This immediate reward evaluates the quadruped’s behavior and progress toward task accomplishment and guides learning so that appropriate learning takes place. The reward function is computed according to the following equation:

(4) $$r=r_{g}+r_{lv}+r_{av}+r_{b}+r_{fd}$$

where $r_{g}$ is a reward term that evaluates reaching the goal, $r_{lv}$ is a reward term that evaluates the degree of following the target movement speed, $r_{av}$ is a reward term that evaluates the degree of following the target rotation speed, $r_{b}$ is a reward term that calculates the penalty for deviating movement to the target movement speed and target rotation speed, and $r_{fd}$ is a reward term that calculates the penalty for falling over. These are added together and used as the immediate reward. The detailed calculation of each reward term is shown.

#### 6.2.2. Reward for Reaching Goal: $r_{g}$

The $r_{g}$ is expressed as

(5) $$r_{g}=\left\{\begin{array}{c} g,\;p_{r}\in GoalBoundingBox\\ 0,\;otherwise \end{array}\right.$$

and it evaluates whether the robot has reached the goal region (*GoalBoundingBox*). $p_{r}$ indicates the current position of the robot, and a higher reward g is obtained if this position is within the goal region. g is set to a value of 10,000 to emphasize that reaching the goal is the final goal of the task. The reward g is obtained only when the goal is reached; otherwise, it is zero. With this reward item, the robot is motivated to learn behaviors to reach the goal.

#### 6.2.3. Reward for Tracking Movement Speed: $r_{lv}$

The $r_{lv}$ is expressed as

(6) $$r_{lv}=\left\{\begin{array}{c} \exp\left(-2.0{\left(v_{pr}-0.6\right)}^{2}\right),\;v_{pr}<0.6\\ 1.0,\;v_{pr}\geq 0.6\\ 0.0,\;zero\;command\; \end{array}\right.$$

and it evaluates the degree of tracking of the robot’s actual speed ($v_{xy}$) relative to the target movement speed $\left(v_{T_{xy}}\right)$. Here, $v_{pr}$ is the inner product of $v_{T_{xy}}$ and $v_{xy}$, which quantifies the degree of tracking. In this study, the calculation is divided into cases depending on whether $v_{pr}$ is greater than or equal to 0.6, less than 0, or 0, in reference to the previous research [20].

If $v_{pr}$ is greater than 0.6, 1.0 is returned as the case of good tracking. If $v_{pr}$ is greater than 0 and less than 0.6, the calculation is made exponentially smaller in the range of 0~1, and 0 is returned in the case of 0.

#### 6.2.4. Reward for Tracking Rotation Speed: $r_{av}$

The $r_{av}$ is expressed as

(7) $$r_{av}=\left\{\begin{array}{c} \exp\left(-1.5{\left(\omega_{pr}-0.6\right)}^{2}\right),\;\omega_{pr}<0.6\\ 1.0,\;\omega_{pr}\geq 0.6 \end{array}\right.$$

and it evaluates the degree to which the robot’s actual rotation speed ω tracks the target rotation speed $\omega_{T}$. Here, $\omega_{pr}$ is the inner product of the two and represents how closely the robot follows the target rotational speed. This reward term allows the robot to learn more precise directional and rotational control.

#### 6.2.5. Moving and Rotating Deviation Penalty: $r_{b}$

The $r_{b}$ is expressed as

(8) $$r_{b}=\exp\left(-1.5v_{0}^{2}\;\right)+exp(-1.5||\omega_{xy}|{|}^{2})$$

and it is calculated based on the divergence between the target speed and the actual speed $v_{0}$ and the angular velocity in the xy-plane $\omega_{xy}$. This reward term penalizes the robot when it makes unnecessary movements or unnecessary rotations. This encourages the robot to move more efficiently and purposefully. Here, $v_{0}$ is calculated as $\left||v_{xy}-v_{pr}\cdot v_{T_{xy}}|\right|$.

#### 6.2.6. Penalty for Falling Down: $r_{fd}$

The $r_{fd}$ is calculated as

(9) $$r_{fd}=\left\{\begin{array}{c} 0,\;{rot}_{min}<rot_{x},rot_{y},rot_{z}<rot_{max}\;\\ f,\;otherwise \end{array}\right.$$

and it is the penalty given if the robot falls down, with a penalty f (set to −1000) if the robot’s tilt angle $\left(rot_{x},\;rot_{y},\;rot_{z}\right)$ exceeds the acceptable range. With this reward term, the robot learns to perform the task while maintaining stability.

### 6.3. Experimental Results 1

**Figure 25:**
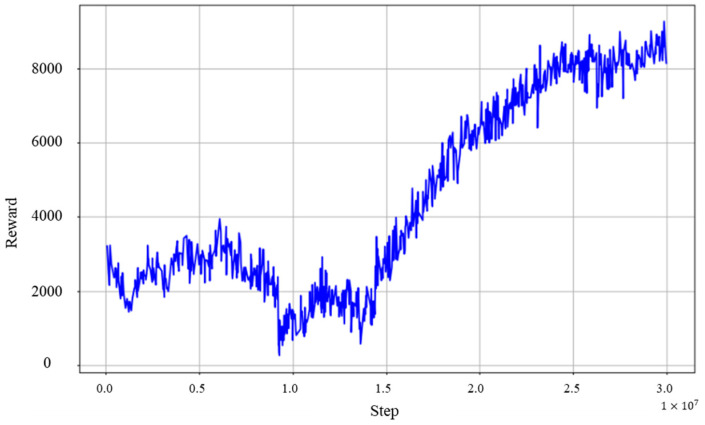
Reward graph in the stair-ascending motion acquisition experiment.

The results of the stair-climbing acquisition experiment are as follows: Reinforcement learning took place over 30 million steps. The reward graph shown in Figure 25 indicates that the learning process reached its final curriculum near 15 million steps. While there was an increase in rewards as the curriculum progressed, there were temporary drops in the reward graph as the target achievement criteria for each curriculum step were reached. This may indicate a transition in learning due to the adaptation to the new curriculum phase.

Using the model at the completion of the learning phase, the success rate was calculated and a value of 0.95 was obtained, as 95 successes were seen out of 100 trials. This indicates the effectiveness of the learning approach and curriculum design in this research, which achieved a high success rate in the stair-climbing task with the assumption of human onboarding.

### 6.4. Discussion

Analysis of the learning process in this experiment revealed that the sequential progression of curriculum learning contributed significantly to the final success rate. The goal accomplishment criteria set at each curricular stage enabled the robot to adapt to complex tasks in a sequential and efficient manner, underscoring the importance of reward design. In particular, setting high rewards for goal accomplishment reinforced motivation in task execution and contributed to the efficiency of the learning process. The success rate of the final learning model reached 0.95, indicating that the approach used in this study was highly effective in the stair-climbing task. This high success rate means that the design of the learning process, especially the optimization of the curriculum progression method and reward structure, led to efficient and effective learning outcomes.

### 6.5. Experiment for Acquiring Behavior in Staircase Descent

**Figure 26:**
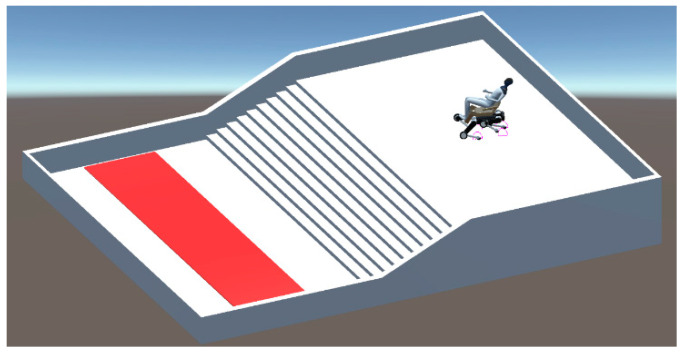
Field used in stair-descending motion acquisition experiment.

In the stair descent acquisition experiment, the same staircase as in the ascent experiment was used. In this environment, a quadruped wheelchair with a person on board is placed on the second floor, and a goal area is set up on the first floor as shown in Figure 26. The goal area is marked with a red strip, and the quadruped wheelchair is considered to have reached the goal when it reaches this area. The path is generated using NavMesh and calculated to head toward the center of gravity of the goal area. In the experiments, PMTG is used for gait control and PPO is used as the learning algorithm, which promotes stable learning and efficient policy improvement. Since the direct implementation of the stair descent behavior would make it difficult to achieve the goal, it is essential to design an appropriate curriculum. The curriculum will be similar to that used in the ascent experiment and will be adjusted for distance from the wheelchair to the goal area, the kick height, and the passenger’s body weight. The same reward functions as in the ascent experiment will be used.

### 6.6. Experimental Results 2

**Figure 27:**
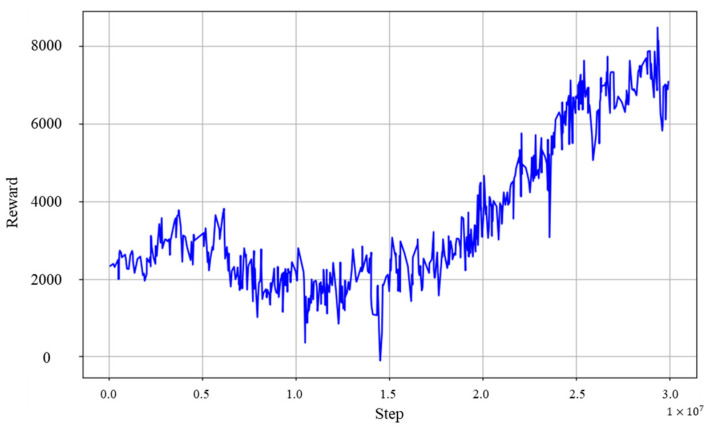
Reward graph in stair-descending motion acquisition experiment.

Figure 27 shows a graph of the learning results of the stair descent acquisition experiment. It can be seen that learning progressed smoothly overall, reaching the final curriculum in approximately 15 million steps, after which the reward sum gradually increased. Interestingly, in comparison to the stair ascent acquisition experiment, the final values at reward increase and convergence were somewhat smaller, even though the same reward functions were used. This difference could be attributed to the inherent difficulty and characteristics of the stair descent movements. Furthermore, the success rate of the model applied after the end of the training was calculated to be 0.91, as 91 successes were observed out of 100 trials. This success rate may reflect the fact that the stair descent movement is more complex than the stair ascent movement, and in particular, it is more difficult to maintain balance and stability.

## 7. Concluding Remarks

The purpose of this study is to develop a transformable quadruped wheelchair to overcome the mobility barriers of stairs and steps faced by wheelchair users. The proposed quadruped wheelchair is designed based on the Unitree B2, a commercial quadruped robot. The wheelchair uses wheels to move on flat ground and legs to move on stairs and steps. It is also equipped with hemispheric 3D LiDAR on its left and right sides, IMU on its torso, and force sensors on its toes, enabling it to recognize its surroundings and move around.

In the experiment, the Unity game engine was used to construct an environment that simulated the robot’s movements and physical interactions, and reinforcement learning was used to help the robot learn the stair-climbing motion of a quadruped wheelchair. Curriculum learning was employed as part of the reinforcement learning, and the robot learned the movements by increasing the difficulty of the task step by step. As a result, the quadruped wheelchair was able to ascend and descend stairs even with a person in it and achieved a success rate of 0.95 in the stair-climbing acquisition experiment and 0.91 in the descent acquisition experiment.

## 8. Future Work

A future challenge is to develop a quadruped wheelchair that can handle a greater variety of stairs and environments. Current quadruped wheelchairs can handle specific stairs and steps but not complex stairs such as spiral stairs, for example. Therefore, further experimentation and improvement is needed. In addition, the current success rate of quadruped wheelchairs remains inadequate with respect to safety, pointing to the need to improve stability and safety, especially in the descent motion. This requires further improvement of the gait control algorithm and structural improvement of the quadruped mechanism itself. This study proposed a new approach to solving the mobility barriers faced by wheelchair users and showed the potential for its practical application and technological advancement. The approach is expected to provide more freedom of movement to more people in the future.

The source codes for the quadruped wheelchair simulator and machine learning model developed in this study, along with a demonstration video, are provided in the Supplementary Materials as indicated below.

## Figures and Tables

**Table 1:** Scope of each parameter.

Parameter	Minimum Value	Title 3
Kick height	0.01 cm	12 cm
Distance to goal Weight of passenger	0.1 m 10 kg	7 m 57 kg

**Figure A1:**
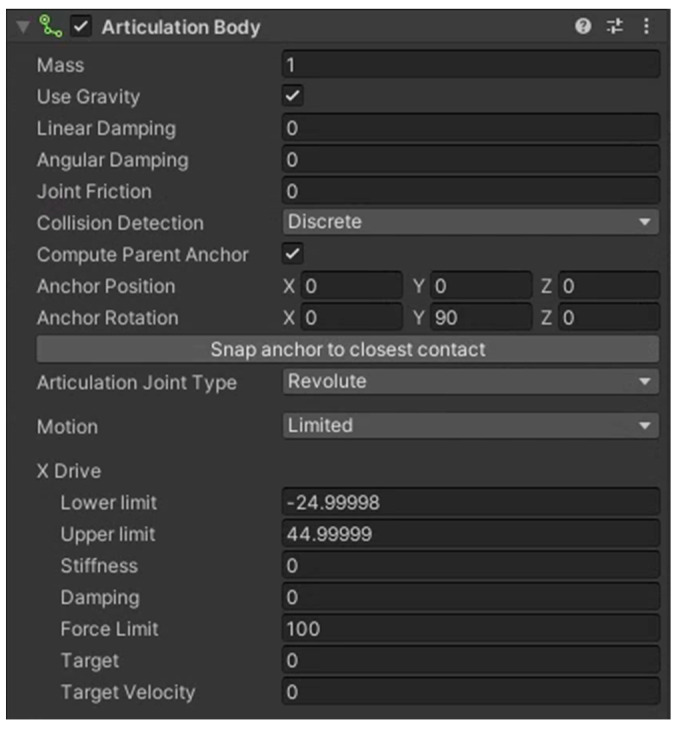
ArticulationBody component.

**Figure A2:**
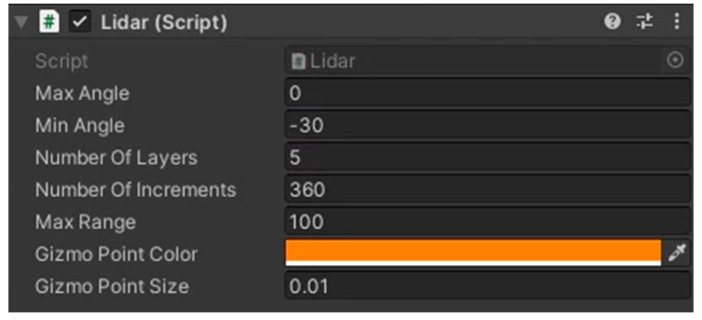
LiDAR component provided by VTC on Unity.

## Data Availability

The data used in this paper were generated by a simulator. Therefore, the source code of the simulator was released as a substitute for the data.

## Supplementary Materials

The following are available online: source codes of machine learning at https://github.com/AkamisakaAtsuki/transformable_quadruped_wheelchair_unity (accessed on 20 April 2024), and video demonstration at https://youtu.be/OFdYSUuzGrQ (accessed on 20 April 2024).

## Appendix A. Actuator Implementation

The actuator is produced by Unity’s ArticulationBody component, which combines the traditional RigidBody and Joint components into a single component. In the past, the simulation of joints was inaccurate, causing frequent problems with parts being misaligned or missing during operation, but with the introduction of ArticulationBody, these problems have been resolved and a more accurate simulation is now possible.

The ArticulationBody component is attached to the object through the Inspector. As shown in Figure A1, the parameters listed for Articulation Joint Type, Motion, and X Drive are important for properly configuring the actuator. These include Lower limit, Upper limit, Stiffness, Damping, Force Limit, Target, and Target Velocity. Articulation Joint Type sets whether the joint is Fixed, Revolute, Prismatic, or Spherical. In many robots, the joints are often the Revolute type. Motion sets restrictions on the joints if needed. For example, if the joint is a Revolute type, selecting “Limited” disables infinite rotation, while selecting “Free” enables infinite rotation. Lower limit indicates the lowest angle at which the joint can operate, and Upper limit indicates the highest angle at which it can operate. Stiffness is P for PD control, and Damping is D for PD control. Force Limit indicates the maximum force the actuator can exert, Target indicates the target position, and Target Velocity indicates the target velocity.

Attaching ArticulationBody components and setting their values is automated by using the URDF Importer in the Unity Robotics Hub, but manual settings are required for Stiffness, Damping, and Force Limit. Because quadruped robot actuators require high torque and responsiveness, experiments are often conducted based on assumptions of an ideal actuator. In this research, the values given in the Unity Robotics Hub tutorial, specifically Stiffness of 10,000, Damping of 100, and Force Limit of 1000, were used for the experiments on the quadruped wheelchair.

## Appendix B. Sensor Implementation

### Appendix B.1. LiDAR Component Provided by VTC on Unity

What follows details the creation of the various sensors used in the quadruped wheelchair, in which hemispherical 3D LiDAR, IMU, and force sensors play important roles. First, hemispherical 3D LiDAR is explained. The package VTC on Unity (Virtual Tsukuba Challenge on Unity) [49] is very useful for LiDAR simulation.

The component that realizes LiDAR will look like Figure A2 when it is introduced into the Unity editor. Through this component, the angular range of the 3D LiDAR in the tilt direction (Max Angle and Min Angle), the number of layers (Number Of Layers), the resolution in the rotational direction (Number Of Increments), and the maximum measurable distance (Max Range) can be set. For an accurate setting of a hemispherical 3D LiDAR such as Robosense’s Bpearl used in this research, Max Angle should be set to set to 0 degrees and Min Angle to -90 degrees (or vice versa).

Also, Number Of Layers should be set to 32, Number Of Increments to 360, and Max Range to 100. However, since these settings increase the computational cost and greatly reduce the simulation speed of Unity, we decided to set the measurement range and resolution smaller, as shown in Figure A2. When the actual device is used, this can be handled by extracting the relevant portions from the measured data. The sensor provided by VTC on Unity has a function to add Gaussian noise to the measured values, but this function was not used in this experiment.

### Appendix B.2. IMU Implementation

The IMU program records the position and rotation angle data obtained from Transform, as well as the time data showing when the function was executed, and derives the velocity, acceleration, and angular velocity from these data. Since no Gaussian noise was added to these data, the data were used as they were measured.

### Appendix B.3. Force Sensor Implementation

For the force sensor, a script called “ToeCollisionDetector” was created and attached to the toe. This script is inherited from the MonoBehaviour class used in the Unity game development environment and is designed to detect the force generated when the toe comes into contact with another object. The class consists of three main elements: the toeForce, the attachedPart, and the child object’s meshCollider. Force measurement is performed by the OnCollisionStay method during ongoing physical contact. This method is called repeatedly during the duration of the contact and is only processed if the object in contact is not an attachedPart. The forces at each contact point are calculated using the normals of the contact points, the relative velocities, and the masses of the contacting objects, and these forces are summed to obtain the final force vector.
